# Real-Time Monitoring of Placental Oxygenation during Maternal Hypoxia and Hyperoxygenation Using Photoacoustic Imaging

**DOI:** 10.1371/journal.pone.0169850

**Published:** 2017-01-12

**Authors:** Chloé J. Arthuis, Anthony Novell, Florian Raes, Jean-Michel Escoffre, Stéphanie Lerondel, Alain Le Pape, Ayache Bouakaz, Franck Perrotin

**Affiliations:** 1 Inserm U930, François Rabelais University, Tours, France; 2 University Hospital Center of Tours, Department of Obstetrics, Gynecology and Fetal Medicine, Tours, France; 3 PHENOMIN-TAAM-UPS44, Center for Small Animal Imaging (CIPA), CNRS Orléans, France; Otto von Guericke Universitat Magdeburg, GERMANY

## Abstract

**Purpose:**

This preclinical study aimed to evaluate placental oxygenation in pregnant rats by real-time photoacoustic (PA) imaging on different days of gestation and to specify variations in placental oxygen saturation under conditions of maternal hypoxia and hyperoxygenation.

**Material and methods:**

Placentas of fifteen Sprague-Dawley rats were examined on days 14, 17, and 20 of pregnancy with a PA imaging system coupled to high-resolution ultrasound imaging. Pregnant rats were successively exposed to hyperoxygenated and hypoxic conditions by changing the oxygen concentration in inhaled gas. Tissue oxygen saturation was quantitatively analyzed by real-time PA imaging in the skin and 3 regions of the placenta. All procedures were performed in accordance with applicable ethical guidelines and approved by the animal care committee.

**Results:**

Maternal hypoxia was associated with significantly greater decrease in blood oxygen saturation (ΔO_2_ Saturation) in the skin (70.74% ±7.65) than in the mesometrial triangle (32.66% ±5.75) or other placental areas (labyrinth: 18.58% ± 6.61; basal zone: 13.13% ±5.72) on different days of pregnancy (*P*<0.001). ΔO_2_ Saturation did not differ significantly between the labyrinth, the basal zone, and the decidua. After the period of hypoxia, maternal hyperoxygenation led to a significant rise in oxygen saturation, which returned to its initial values in the different placental regions (*P*<0.001).

**Conclusions:**

PA imaging enables the variation of blood oxygen saturation to be monitored in the placenta during maternal hypoxia or hyperoxygenation. This first preclinical study suggests that the placenta plays an important role in protecting the fetus against maternal hypoxia.

## Introduction

*In vivo*, fetal oxygenation results from transplacental oxygen transfer from the maternal to the fetal vascular compartment. Impaired oxygen delivery to the fetus may be observed in pathological conditions including placental insufficiency and maternal chronic hypoxia and carries the risk of such severe fetal consequences during the prenatal period as intrauterine growth restriction (IUGR), metabolic acidosis, and fetal death [1]. It is thus essential to be able to evaluate the impairment of placental function and oxygenation in clinical practice, especially in pathological situations. The reference methods today for placental insufficiency screening and follow-up are Doppler ultrasound and fetal heart rate monitoring [2]. However, neither can be used to measure placental oxygen saturation in clinical practice. Although cordocentesis allows an accurate and reliable measurement of fetal blood oxygenation, the high morbidity associated with this invasive technique makes it useless in clinical practice for placental insufficiency monitoring [3]. Currently, blood oxygen level-dependent magnetic resonance imaging (BOLD MRI) has been suggested to be reliable tools for non-invasive exploration of placental perfusion and oxygenation [4–5].

More recently, photoacoustic (PA) imaging is present as a real-time noninvasive imaging method combining laser pulse tissue excitation and ultrasonic detection of the tissue response [6]. Typically, the ultrasound image is obtained by irradiating tissue with a nanosecond pulsed laser. Optical absorption induces a rapid thermoelastic tissue expansion that generates a wideband ultrasound wave detectable by an ultrasound transducer. The main advantage of this technology is that the optical contrast is combined with the penetration of ultrasound able to detect a backscattered signal at depths up to few centimeters. Indeed, exploration depth depend on the wavelength used for the PA signal. In vivo, tissue contains endogenous chromophores (*e*.*g*., hemoglobin, melanin, and lipids) that can generate detectable PA signals. PA imaging, which uses hemoglobin signals, has been used mainly to image variety of tumors and the blood vasculature [7–8]. Furthermore, spectroscopic PA imaging allows *in vivo* assessment of oxygen saturation of the blood by exploiting the differing optical absorption spectra of deoxyhemoglobin (Hb) and oxyhemoglobin (HbO_2_). Due to limited penetration depth related to the wavelength used for chromophore excitation, PA is currently restricted in clinical practice but appears to be an excellent tool for understanding aspects of placental function in research. The combined imaging of PA and MRI would provided complementary information.

Our objectives in this study were to evaluate placental oxygen saturation in pregnant rats by real-time photoacoustic (PA) imaging during the 3^rd^ week of gestation and to specify the variations in placental oxygen saturation during maternal hypoxia and hyperoxygenation.

## Materials and Methods

### Animal preparation

All procedures were performed in accordance with French and international ethical guidelines and were approved by the National Committee for Animal Care and Ethics in Animal Experiments (No 1037 CECCO n°3). Fifteen pregnant (after timed mating, designated as embryonic day 0) Sprague-Dawley rats (CERJ, Le Genest Saint-Isle, France) were housed in a temperature-controlled room (23°C) with a 12:12-h light-dark cycle and, food and water *ad libitum*.

The rats were anesthetized with 1.5% isoflurane (Aerrane^®^, Baxter France), administered with 1.5 L/min of air via a facial oxygen mask. The anesthetic gas was administered for no more than 1 h and a thermostatically controlled pad was used to maintain body temperature at 37°C. Before PA imaging began, rat abdomens were shaved and a warm, centrifuged ultrasound gel was applied between the transducer and abdomen skin to enhance PA transmission.

### Photoacoustic imaging procedure

**Fig 1:**
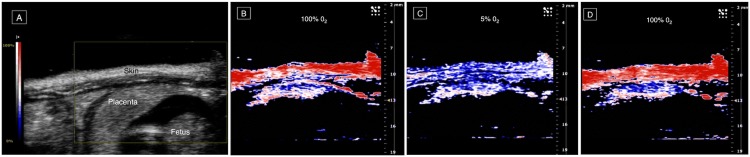
Photoacoustic (PA) imaging of placental oxygenation on day 14 of gestation. From left to right: (A) the placenta in a sagittal plane (obtained by a B-mode ultrasound scan) and parametric images created with the PA Oxyhemo mode making possible the evaluation of blood oxygen saturation during variations in the oxygen levels supplied to the mother (5–100%). PA imaging sequences during hyperoxygenation (B), hypoxia (C) and hyperoxygenation (D).

Transabdominal PA imaging of placentas was performed on days 14, 17, and 20 of gestation. Day 14 was defined as the time required to differentiate the basal and labyrinth zones. The monitoring was performed every 3 days in order to limit the effects of a repeated anaesthesia on the rat gestation. PA images were acquired with a 21 MHz probe (LZ-250; 75 μm axial resolution) connected to the VevoLAZR PA imaging system (Fujifilm Visualsonics Inc., Toronto, Canada). For each high-resolution scan, light from the laser (OPO pumped by doubled Nd:YAG, tunable 680–970 nm, 20 Hz repetition rate, 5 ns pulse width, 50 mJ pulse energy) was delivered to the most superficial placentas, which were positioned at the optical focus between 10 and 15 mm. The Vevo imaging station allows adjustment of the probe and imaging optimization to reduce motion artifacts. The parametric images from which we estimated oxygen saturation levels were displayed by collecting the data at wavelengths of 750 nm and 850 nm in the Oxyhemo mode. Changing the O_2_ concentration in the gas supplied to the mother exposed the fetus successively to 2.5 min at 100% O_2_ inhalation (hyperoxygenation), then 4 min at 5% O_2_ inhalation (hypoxia), and finally 4 min at 100% O_2_ inhalation (hyperoxygenation) for each procedure. PA imaging sequences were recorded at 0.8 frames per second to study tissue oxygenation (Fig 1). No more than 3 procedures were performed per day per animal.

Placental PA imaging was performed in a sagittal plane (Fig 1A). The plane of the skin was used as a reference because the distance from the skin to the probe was similar in each measurement, and the attenuation was minimal. Three distinct parts of each placenta were analyzed: the mesometrial triangle (that corresponds to maternal tissue invaded by fetal trophoblasts) which connects the uterine horn and the placenta; and two placental zones: the labyrinth, closer to the fetus (fetal part), and the basal zone that corresponded to the trophospongium (closer to the maternal part). The labyrinth is the exchange zone of the placenta where nutrients and gases are transferred between the mother and the fetus. The basal zone, also called the trophospongium, is located close to the mesometrial triangle and corresponds to the junction between the labyrinth and the decidual area. This zone is composed of trophoblast stem cells, trophoblast giant cells, and glycogen cells.

**Fig 2:**
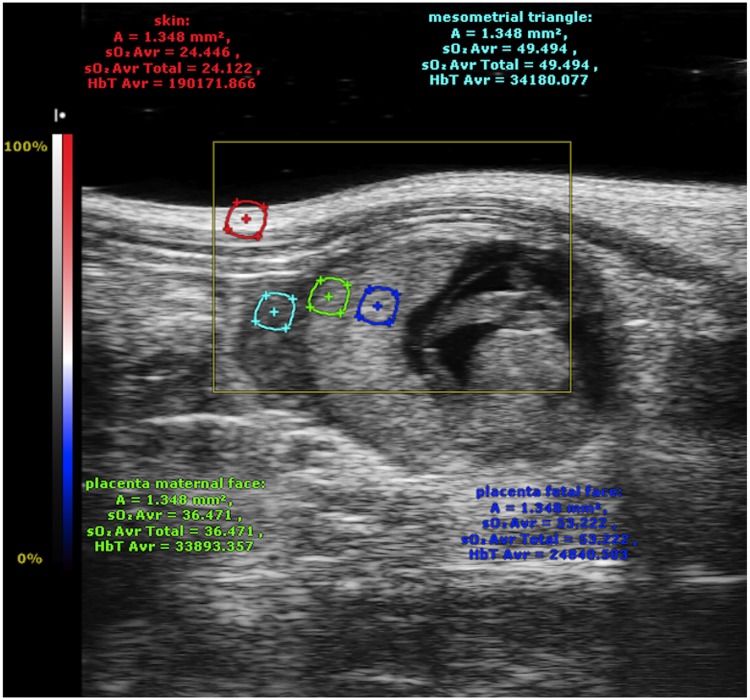
Regions of interest (ROIs) placed on the skin (red), the mesometrial triangle (light blue), and the two different zones of the placenta: the basal zone (green) and the labyrinth zone (dark blue).

After image acquisition, tissue oxygen saturation was quantitatively analyzed from the linear raw data with VevoLAZR software (Fujifilm Visualsonics Inc., Toronto, Canada). Four regions of interest (ROIs) (1.35 mm^2^ diameter, about 11 mm in depth) were manually defined by a single operator (C-J.A): skin, mesometrial triangle, basal zone, and the labyrinth (Fig 2). Time-intensity curves for each ROI were computed with VevoLAZR software. Oxygenation parameters, including oxygen saturation variation during hypoxia (Fig 1C) and hyperoxygenation (Fig 1B and 1D) sequences were calculated for each ROI from the time-intensity curves. Rates of oxygen saturation decrease and oxygen saturation rise were determined from the slope of the fitted curve. ΔO_2_ saturation corresponded to the change in oxygen saturation between hyperoxygenated (100% O_2_ supplied to mother) and hypoxic (5% O_2_) conditions and was defined as follows:

$${\Delta \text{O}}_{\text{2}}\text{saturation }=\;{\text{oxygen saturation}}_{\text{hyperoxygenation}}\;-\;{\text{oxygen saturation}}_{\text{hypoxia}.}$$

On day 20, rats were euthanized by intravenous pentobarbital injection (150 mg/kg) after the imaging procedure.

### Statistical analysis

Statistical analysis was performed with R statistical software (version 3.1.1). Quantitative variables are presented as means ± standard deviations. When more than one placenta was tested on the same rat, the mean value of these nested measurements was taken into account. The paired Friedman test was used to compare the continuous variables between the four ROIs, and the unpaired Kruskal-Wallis test to compare continuous variable between the three gestational days. In cases of significant differences, pairwise comparisons were tested with the Wilcoxon test. Because of the limited sample size, the analysis was restricted to the pairwise combinations of relevant variables. A *P* value < 0.05 was considered to indicate a significant difference.

## Results

The study included 15 rats: five for each day of gestation considered. A total of 9 placentas were studied on days 14 and 17 of gestation, and 12 on day 20. PA imaging was performed on all animals under hyperoxygenated and hypoxic conditions (Fig 1). All time-intensity curves were recorded for about 10 minutes 30 seconds and allowed us to assess the variations in oxygen saturation in the skin and the placenta.

Oxygen saturation variations observed in real-time during PA imaging were quantitatively analyzed with LAZR software (Table 1) at a depth of up to 1.6 cm. During the procedure, variations in oxygen saturation could be clearly visualized according to the maternal oxygenation level.

**Table 1:** Quantitative analysis of oxygenation in the skin and the 3 regions of interest (ROIs) of the placenta, on days 14, 17 and 20 of gestation (pooled data). Results are presented as mean ± standard deviation.

ROI	Day 14 (Mean ± Standard Deviation)	Day 17 (Mean ± Standard Deviation)	Day 20 (Mean ± Standard Deviation)	*P* Value Between day 14–17	*P* Value Between day 17–20	*P* Value Between day 14–20
**Skin**						
Oxygen saturation maximal (%)	84.81 ± 6.72	90.37 ± 5.29	93.17 ± 5.06	**0.07**	**0.34**	**0.007**
Oxygen saturation minimal (%)	18.55 ± 2.22	19.63 ± 5.88	24.63 ± 6.44	**0.86**	**0.09**	**0.007**
Rate of oxygen saturation decrease (%/s)	0.76 ± 0.15	0.88 ± 0.39	0.79 ± 0.13	**0.62**	**0.96**	**0.69**
Rate of oxygen saturation rise (%/s)	1.54 ± 0.59	1.44 ± 0.85	1.08 ± 0.57	**0.73**	**0.23**	**0.05**
**Mesometrial triangle**						
Oxygen saturation maximal (%)	69.98 ± 6.86	70.21 ± 8.84	70.43 ± 8.36	**0.93**	**0.93**	**0.93**
Oxygen saturation minimal (%)	39.23 ± 6.35	37.55 ± 5.44	54.11 ± 5.25	**0.66**	**0.003**	**0.39**
Rate of oxygen saturation decrease (%/s)	0.33 ± 0.10	0.43 ± 0.31	0.23 ± 0.13	**0.92**	**0.08**	**0.07**
Rate of oxygen saturation rise (%/s)	0.61 ± 0.24	0.70 ± 0.64	0.26 ± 0.26	**0.86**	**0.01**	**0.02**
**Labyrinth**						
Oxygen saturation maximal (%)	76.78 ± 4.17	68.21 ± 6.85	65.78 ± 4.94	**0.01**	**0.48**	**0.001**
Oxygen saturation minimal (%)	53.44 ± 1.58	54.63 ± 3.32	54.31 ± 4.25	**0.54**	**0.93**	**0.796**
Rate of oxygen saturation decrease (%/s)	0.28 ± 0.11	0.19 ± 0.13	0.11 ± 0.10	**0.07**	**0.18**	**0.01**
Rate of oxygen saturation rise (%/s)	0.57 ± 0.24	0.23 ± 0.23	0.09 ± 0.05	**0.01**	**0.53**	**0.001**
**Basal Zone (Trophospongium)**						
Oxygen saturation maximal (%)	65.42 ± 10.42	54.80 ± 5.88	55.88 ± 3.59	**0.03**	**0.66**	**0.024**
Oxygen saturation minimal (%)	38.57 ± 3.68	41.67 ± 5.21	44.05 ± 4.57	**0.19**	**0.38**	**0.003**
Rate of oxygen saturation decrease (%/s)	0.31 ± 0.09	0.18 ± 0.08	0.09 ± 0.03	**0.03**	**0.01**	**0.001**
Rate of oxygen saturation rise (%/s)	0.57 ± 0.24	0.18 ± 0.17	0.08 ± 0.04	**0.06**	**0.14**	**0.003**

**Fig 3:**
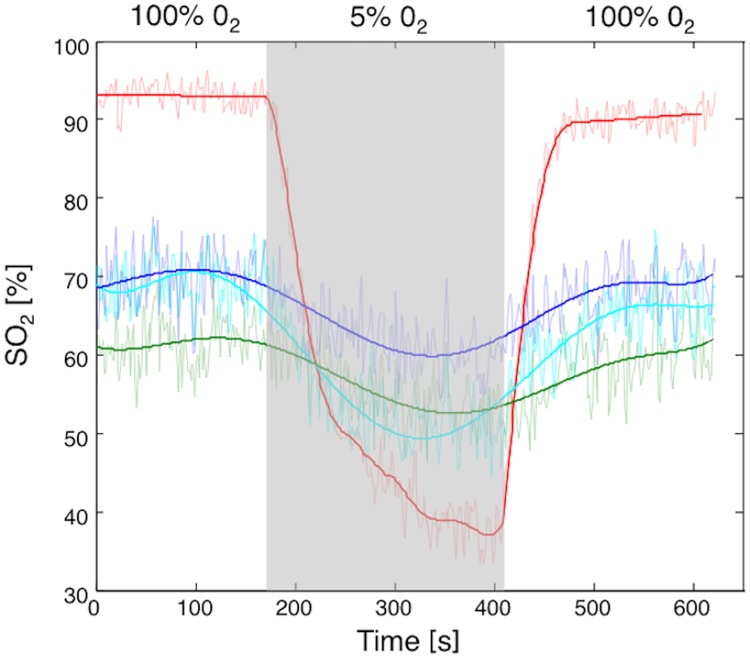
Time-intensity curves obtained during the experimental sequence of hyperoxygenation (100% oxygen, 2.5 min), hypoxia (5% oxygen, 4 min), and hyperoxygenation (100% oxygen, 4 min) from skin (red), mesometrial triangle (light blue), basal zone (green), and labyrinth zone (dark blue) on day 20 of gestation.

A few minutes after O_2_ in the air supplied to the mother was reduced from 100% to 5% (hypoxia), oxygen saturation decreased significantly in each ROI (*P*<0.001). The effect of hyperoxygenation from 5% to 100% inhaled O_2_ was also well visualized by a significant oxygen saturation increase in all four ROIs (*P*<0.001) (Fig 3). Over the course of gestation, from day 14 to day 20, the mean oxygen saturation values of the skin increased (*P* = 0.007) while it decreased in the labyrinth zone (*P*<0.001) and in the basal zone (*P* = 0.02). The rates of oxygen saturation variations during maternal hypoxia and hyperoxygenation (*i*.*e*., the slopes of oxygen saturation decrease and increase corresponding to hypoxia and hyperoxygenation conditions, respectively) were calculated from the raw data. Oxygen saturation rates fell to lower levels in the placenta than in the maternal skin and the mesometrial triangle, and progressively decreased over the course of gestation from day 14 to day 20 (basal zone *P* = 0.003; labyrinth zone P = 0.001). On the other hand, we observed a rapid and deep variation of the oxygen saturation level in the skin under both hypoxic and hyperoxygenated conditions. After hypoxia, oxygen saturation returned to its initial value in about 1 min 20 sec for each ROI.

**Fig 4:**
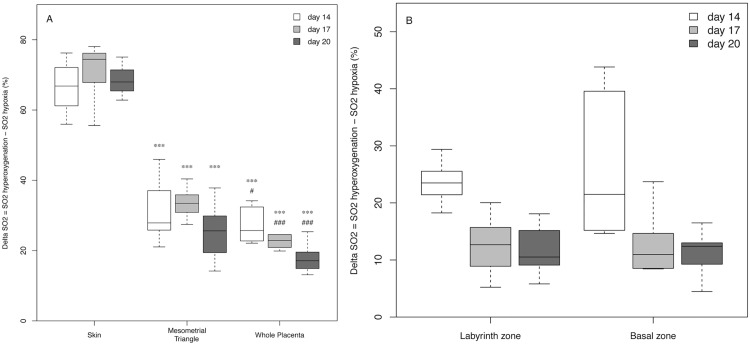
Box-and-whisker plot showing ΔO_2_ corresponded to the mean change in oxygen saturation between hyperoxygenated (100% oxygen supplied to mother) and hypoxic (5% oxygen supplied) saturation. (A) Represents maternal skin, the mesometrial triangle, and the whole placenta on different days of gestation. (B) Box-and-whisker plot represents the different part of the placenta**:** the basal zone and the labyrinth zone on different days of gestation. Data are mean values and 95% confidence intervals. Comparisons between skin and utero-placental regions of interest (mesometrial triangle and whole placenta) at the same day of gestation are indicated by *P = 0.05 **P = 0.01 ***P<0.001. Comparisons between mesometrial triangle and whole placenta at the same day of gestation are indicated by #P = 0.05 ## P = 0.01 ###P<0.001.

Our comparative study thus showed that hypoxia resulted in significant differences in ΔO_2_ saturation between maternal skin and the other ROIs (*P*<0.001) (Fig 4A), with ΔO_2_ saturation higher in the skin (*e*.*g*., 70.74% ± 7.65 on day 17) than in the mesometrial triangle (*e*.*g*., 32.66% ± 5.75 on day 17) or in the different areas of the placenta (*e*.*g*., labyrinth zone: 18.58% ± 6.61; basal zone: 13.13% ± 5.72 on day 17) throughout gestation. In addition, ΔO_2_ saturation decreased in the placenta over the course of gestation (23.34% ± 3.75 on day 14 vs 10.10% ± 4.28 on day 20 in the labyrinth) (*P* = 0.005), at a rate not significantly different between the two placental layers (Fig 4B). These results show that oxygen saturation variations differed significantly from one tissue to another and depended on the day of pregnancy.

Additionally, in order to evaluate the influence of skin attenuation during the procedure, the experiment was repeated after a small surgical incision of the skin that allowed us to image the placenta directly. No significant difference was observed between the two experimental sets (*P*>0.05). These results suggested that the skin has a negligible influence on the PA imaging results.

## Discussion

Our study demonstrates that PA imaging is a useful tool that can be applied to placental oxygen saturation monitoring during hypoxic condition in pregnant rats. Moreover, our experiment suggests that, during maternal hypoxia, the decrease in oxygen saturation affects the placenta less than it affects maternal tissues (*i*.*e*., skin and mesometrial triangle).

The placenta’s apparent ability to supply oxygen during maternal hypoxia may be explained on one side by the high capillary network, which increases during the course of gestation, and on the other hand by an increase of the blood flow. Moreover, we recently demonstrated in a study of uteroplacental perfusion with contrast-enhanced ultrasound that the intervillous space fills with maternal blood slowly, continuously, and uniformly [9]. This substantial degree of vascularization may preserve placental oxygenation for a period of time. The level of oxygen saturation in the ROI depends on the relative proportion of the ROI occupied by maternal blood. Therefore, the increase of oxygen saturation at the end of the pregnancy could be attributed to vessel dilatation between days 14 and 17 of gestation. Furthermore, a more developed vascular network could also cause an overall increase in vessel volume per unit volume of tissue. The differences observed between days 17 and 20 is attributed to an increase in blood flow at the end of gestation. Although, the oxygen saturation level in the labyrinth corresponds to the mix of fetal and maternal hemoglobins, it is important to mention that the measurement of oxygen saturation via PA modality in this region remains valid since spectral absorption curves for fetal and adult hemoglobins are similar [10].

Functional ultrasound and MRI are promising tools for exploring placental oxygenation. Several studies have assessed placental oxygenation by BOLD MRI during gestation of rats or sheep exposed to hypoxia and have established that it can reveal changes in fetal blood oxygenation during hypoxia [4,5,11–14]. More recent studies have reported the ability of PA imaging to demonstrate variations in the oxygenation of tumors or tissues [6–8]. To our knowledge, placental oxygenation over the pregnancy has not previously been evaluated by PA imaging. We therefore compare our results with those of studies by functional MR imaging.

Our results confirm that the placenta is sensitive to oxygen variations. Compared to the skin, the placenta is less sensitive to oxygen variations since differences in skin are much higher. This would support the oxygen reserve capacity of the placenta. Different regions within the placenta may be distinguished, according to the specific enhancements made in perfusion imaging and histological analyses [15]. Published studies have found no difference of oxygenation in these different areas [4,12]. Accordingly, the relatively small differences we observed between the basal zone and the labyrinth zone are not significant.

Wedegartner *et al*. [11] used BOLD MRI to compare maternal oxygenation to that of the placenta and fetal organs of six ewes during hypoxia. Authors reported changes in placental saturation smaller than those observed on the skin of the mother, similar to the trends we observed here with PA imaging.

PA imaging and BOLD MRI were recently compared with the use of a tissue-mimicking phantom in multiple oxygenation conditions [16]. The authors found a good correlation between oxygen saturation derived from PA imaging, T2 signal intensity change on BOLD MRI, and PO_2_ measurements in phantoms. They also noted that the PA image was the most sensitive, with a signal increase approximately 25% greater than the changes observed in the BOLD MRI. The resolution of the PA imaging depends on the resolution of the US transducer. In contrast to MRI procedures, PA imaging allows real-time observation of blood oxygen saturation at different levels of oxygenation in the air supplied to the mother. On the other hand, both PA and BOLD MRI are measuring relative percent oxygen saturation and not measuring absolute numbers of oxygen saturation.

Our study nonetheless has some limitations. First of all, we do not compare PA values to PO_2_ measured in the labyrinth space blood sample. Indeed, the catheterization of these vessels was not possible. However, oxygen levels at the output of the respirator were controlled and oxygen variations were monitored by skin signal. Furthermore, an important limitation of PA imaging lies in the reduced light penetration depth due to light attenuation, which hampers the exploration of deep tissues. In our study, the depth of exploration of our PA imaging platform was limited to about 16 mm. Therefore, our study focused on the placenta and not the fetal organs. Previous studies, however, have reported the ability of PA imaging to detect sentinel lymph nodes in rats at an imaging depth of 25 mm to 31 mm [17–18]. Kruger *et al*. designed a clinical system to evaluate breast cancer, with exploration depths in tissues up to 50 mm and a resolution of 0.42 mm [19]. PA signals depend on optical fluence and absorption. Stronger optical fluence or lower resolution will enable PA imaging to detect placental oxygenation levels at even greater depths while maintaining a safe procedure [20]. The change in laser wavelength is one possible option to improve the depth of exploration. Indeed, a 10 cm imaging depth may be reached at 1064 nm wavelength [21]. However, at this wavelength**,** total hemoglobin could be determined but not the oxygen saturation based on the analysis of oxyhemoglobin and deoxyhemoglobin. Thus, the measurement of oxygen saturation using PA in a deep tissue is an ongoing challenge [22]. PA imaging is currently being investigated in preclinical and clinical studies, and some of them show its potential applications in clinical practice [23–25]. In obstetrical practice, the depth of exploration is currently the limiting factor. The placenta is located in positions more or less uniformly distributed around the uterus. If imaging is done trans-abdominally, then the placenta is probably accessible in about a third of the women. Similarly, if transvaginal imaging is performed, one might expect that the placenta would be out of range in about half of the women. In addition, PA will be used more easily on the first trimester of pregnancy because the uterus size is limited. This application would be interesting to screen high-risk pregnancy. Otherwise, another potential clinical application is described in photocoagulation of inter-twin placental anastomosing vessels to treat twin-to-twin transfusion syndrome. PA imaging in combination with ultrasonic tracking could be useful for detecting the human placental vasculature during minimally invasive fetal surgery [26]. FDA clinically approves the use of lasers (Nd:YAG or Diode lasers) in fetal surgery for selective photocoagulation at a power setting of 30–45 W [27]. Here, our procedure was performed with a 1000 times less output power, suggesting its safety.

In conclusion, PA imaging is a real-time, noninvasive method that allowed us to evaluate placental oxygenation without contrast agent injection. Our results demonstrating that placenta is less affected than maternal tissue by the decline in oxygen saturation suggest that this organ may play an important role in protecting the fetus against maternal hypoxia. Future studies are needed to better understand the underlying mechanisms involved in the regulation of placental oxygenation during situations of potential fetal hypoxia.
